# Intersegmental Interactions Give Rise to a Global Network

**DOI:** 10.3389/fncir.2022.843731

**Published:** 2022-02-23

**Authors:** Graciela Kearney, Martina Radice, Agustín Sanchez Merlinsky, Lidia Szczupak

**Affiliations:** ^1^Instituto de Fisiología, Biología Molecular y Neurociencias (IFIBYNE-UBA-CONICET), Buenos Aires, Argentina; ^2^Departamento de Fisiología, Biología Molecular y Celular, Facultad de Ciencias Exactas y Naturales, Universidad de Buenos Aires, Buenos Aires, Argentina

**Keywords:** central pattern generator, motor control, rhythmic motor pattern, intersegmental coordination, recurrent inhibition

## Abstract

Animal motor behaviors require the coordination of different body segments. Thus the activity of the networks that control each segment, which are distributed along the nerve cord, should be adequately matched in time. This temporal organization may depend on signals originated in the brain, the periphery or other segments. Here we evaluate the role of intersegmental interactions. Because of the relatively regular anatomy of leeches, the study of intersegmental coordination in these animals restricts the analysis to interactions among iterated units. We focused on crawling, a rhythmic locomotive behavior through which leeches move on solid ground. The motor pattern was studied *ex vivo*, in isolated ganglia and chains of three ganglia, and *in vivo*. Fictive crawling *ex vivo* (*crawling*) displayed rhythmic characteristics similar to those observed *in vivo*. Within the three-ganglion chains the motor output presented an anterior-posterior order, revealing the existence of a coordination mechanism that occurred in the absence of brain or peripheral signals. An experimental perturbation that reversibly abolished the motor pattern in isolated ganglia produced only a marginal effect on the motor activity recorded in three-ganglion chains. Therefore, the segmental central pattern generators present in each ganglion of the chain lost the autonomy observed in isolated ganglia, and constituted a global network that reduced the degrees of freedom of the system. However, the intersegmental phase lag in the three-ganglion chains was markedly longer than *in vivo*. This work suggests that intersegmental interactions operate as a backbone of correlated motor activity, but additional signals are required to enhance and speed coordination in the animal.

## Introduction

Animal motor behaviors require the coordination of different body segments, which implies that the networks that control each segment are adequately interconnected. It has been clearly established for a wide spectrum of organisms that, while the brain determines the initiation of locomotive behaviors, the body movements are controlled by central pattern generators (CPGs) distributed throughout the vertebrate spinal cord or the chain of invertebrate midbody ganglia (Orlovsky et al., 1999; Buschges et al., 2008; Puhl and Mesce, 2008; Mullins et al., 2011; Mulloney and Smarandache-Wellmann, 2012; Borgmann and Buschges, 2015; Katz, 2015; Fushiki et al., 2016; Kiehn, 2016; Saltiel et al., 2017; David and Ayali, 2021). A central question that remains open is how a global behavior emerges from the concert of local controllers (Buschmann et al., 2015; Lewis, 2016; Saltiel et al., 2017). Because of their simple anatomical structure and their robust motor patterns leeches are an outstanding organism to analyze motor control (Kristan et al., 2005).

**FIGURE 1:**
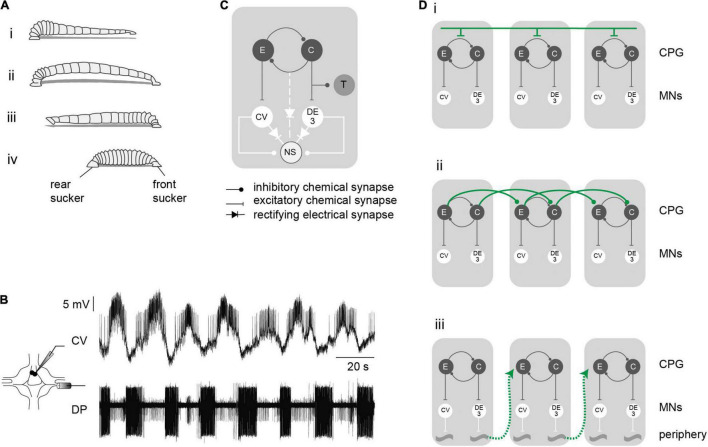
Crawling motor pattern. **(A)** The drawing depicts a leech crawling step that results from coordinated waves of elongation **(i,ii)** and contraction **(iii,iv)** phases. **(B)** *Crawling* is induced in the isolated nervous system by dopamine and can be monitored through intracellular recordings of the CV motoneuron and extracellular recordings of the DE-3 motoneuron in the DP nerve, whose activities correspond to the elongation and contraction phases of crawling, respectively. **(C)** Schematic network interaction underlying crawling. **(D)** Diagrams of different hypothetical pathways controlling intersegmental coordination. Each gray box represents a leech segment bearing a crawling central pattern generator (CPG), constituted by a half-center oscillator (C and E) responsible for the excitation of the motoneurons (MNs) active in each phase. Intersegmental coordination depends on: **(i)**, a command neuron located in the cephalic ganglion that sequentially activates each segmental CPG; **(ii)**, the interaction of local circuits (for simplicity we limited the connections to one direction, but they can operate in both directions); **(iii)**, sensory feedback from the periphery.

The leech body is formed by 21 identical midbody segments, each one controlled by a midbody ganglion. Since the ganglia are highly similar, the question about intersegmental coordination becomes a query on the interactions among iterated units. Among leech locomotive behaviors, crawling is well suited to address this matter. This behavior results from waves of elongation and contraction that propagate along the body segments, as the animal is anchored on the posterior and anterior suckers, respectively (Figure 1A). Fictive crawling (*crawling*) can be monitored in the isolated nervous system (Eisenhart et al., 2000) by recording the alternated activation of motoneurons that innervate circular [e.g., circular ventral (CV) motoneuron] and longitudinal muscles [e.g., dorsal excitor cell 3 (DE-3) motoneuron], with due metachronal order along the cord. Moreover, the basic motor pattern (Figure 1B) also takes place in single isolated ganglia (*crawling-pattern*), indicating that each ganglion contains the network that controls this rhythmic output (Puhl and Mesce, 2008). Here we have investigated the interactions among these putatively autonomous rhythmic modules.

Figure 1C presents a scheme of the rhythmic module underlying the *crawling-pattern* in each ganglion. Previous studies have proposed that crawling is controlled by a half center oscillator (Cacciatore et al., 2000) formed by two groups of neurons that excite the motoneurons active during the elongation and the contraction phases. The identity of the neurons forming the CPG that controls the *crawling-pattern* is currently unknown. The pair of premotor non-spiking (NS) neurons are linked to the motoneurons through chemical and electrical junctions (Wadepuhl et al., 1990; Rela and Szczupak, 2007). In isolated ganglia it was shown that this pair of premotor neurons are linked to the CPG that controls the *crawling-pattern* (Rodriguez et al., 2012). In addition, this segmental CPG projects a reafferent inhibitory signal to mechanosensory touch (T) cells (Alonso et al., 2020).

Previous studies suggest that intersegmental connectivity is provided by three possible pathways: (i) top-down signals delivered by brain neurons that control the segmental delay; (ii) connections among the CPGs of adjacent segments that rule the timing of each CPG; and (iii) the CPGs are coordinated through proprioceptive stimuli caused by specific movements (Figure 1D). For simplicity only short range intersegmental connections are represented in Figures 1Dii,iii. Here, we analyzed whether *crawling* can be induced in short chains of midbody ganglia, deprived of brain and peripheral signals, and compared the output with the *crawling-pattern* exhibited by isolated single ganglia and with the behavior in intact animals. The results indicate that short chains of ganglia exhibited a rhythmic activity comparable to that evoked in single ganglia and to the actual rhythmic behavior *in vivo*. The results uncover that what seemed a chain of autonomous segmental CPGs, functions as a global network that controls the coordination of the motor output.

## Materials and Methods

### Biological Preparation

Leeches (*Hirudo* sp.) weighing 2–5 g, were obtained from commercial suppliers (Niagara Leeches, Cheyenne, WY, United States) and maintained at 15°C in artificial pond water. These animals are hermaphrodites. The leech nervous system is composed of a chain of 21 midbody ganglia flanked by head and tail brains. Each midbody ganglion contains all the sensory and motor neurons that innervate the corresponding segment *via* root nerves (Muller et al., 1981).

Electrophysiological studies were performed in isolated single ganglia or in chains of three ganglia, obtained from midbody ganglion 7–13. The ganglia were dissected with one dorsal posterior (DP) nerve roots attached to it. The tissue was bathed in normal saline (in mM: 115 NaCl, 4 KCl, 1.8 CaCl_2_, 1 MgSO_4_, 10 Hepes, 10 glucose; pH 7.4) at room temperature (20–25°C) and pinned to Sylgard (Dow Corning) in a recording chamber. The sheath covering the ganglion was dissected away, leaving the neuronal cell bodies exposed to the external solution.

### Electrophysiological Recordings

Intracellular somatic recordings were made with microelectrodes pulled from borosilicate capillary tubing (FHC, Brunswick, ME, United States), filled with 3 M potassium acetate (resistance 20–40 MΩ). The electrodes were connected to an Axoclamp 2B amplifier (Axon Instruments; Union City, CA, United States) operating in bridge mode. Extracellular activity was recorded from dorsal posterior (DP) nerves using suction electrodes connected to a differential a.c. amplifier (Neuroprobe 1700, AM-Systems, Inc., Carlsborg, WA, United States). The intra and extracellular recordings were digitized using an analog-digital converter (Digidata 1440, Axon Instruments, Union City, CA, United States) and acquired using a commercial program (Clampex 9.2, Axon Instruments, Union City, CA, United States) at a sampling rate of 5 kHz. The sensory T and premotor NS neurons were readily recognized by their soma location and electrophysiological properties (Nicholls and Baylor, 1968; Rela et al., 2009).

To evoke a rhythmic motor episode the ganglion or the chain were superfused with 75 μM dopamine hydrochloride (Sigma-Aldrich, St. Louis, MO, United States) prepared fresh at the beginning of each experimental day (Puhl and Mesce, 2008). Only one episode was evoked *per* ganglion or chain of ganglia. The rhythmic motor pattern was monitored *via* extracellular recording of the DP nerve, where the largest spike corresponds to the DE-3 motoneuron (Ort et al., 1974). This cell is active during the contraction phase of crawling (Baader and Kristan, 1992; Baader, 1997).

When the effect of NS neurons was tested hyperpolarizing pulses were injected in this neuron in the isolated ganglia or in the middle ganglion of the chain. At least four cycles before and after an inhibitory pulse were left unaffected to obtain pre- and a post-pulse rhythmic epochs.

### Behavioral Experiments

Leeches were anesthetized by placing individuals at −20°C for 30 min. Then the surface of the animal was dried and points of water-based paint were drawn along their dorsal longitudinal axis (Figure 7A). When the animals recovered they were allowed to crawl on a white smooth surface. A camera (Nikon Coolpix s4400), supported above the animal on a wheeled holder, was used to film at 30 fps.

Position of each dot was tracked using an OpenCV based algorithm in Python (RRID:SCR_008394).^1^ The position of the front and rear edge of the leech were computed by calculating the intersection between the animal contour and a linear fit of the first three points with the front edge, and the last three with the rear edge.

### Data Analysis

**FIGURE 2:**
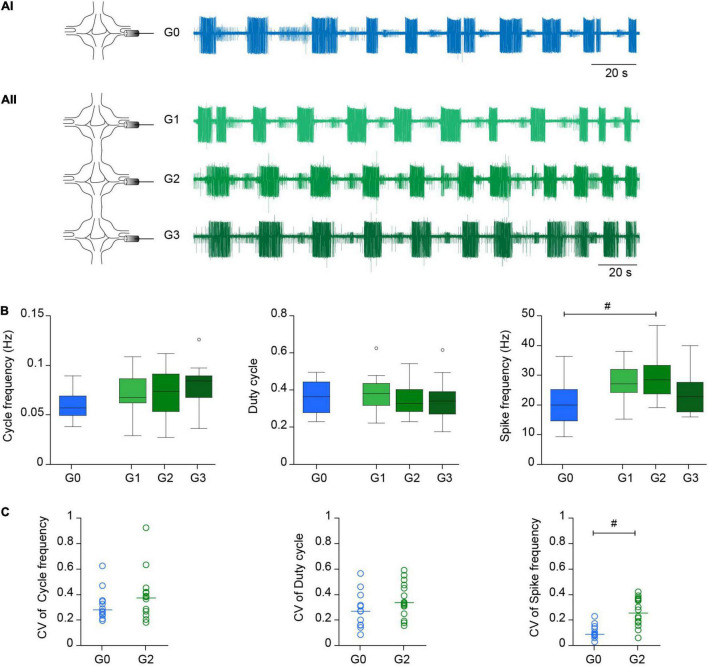
C*rawling* in reduced experimental configurations. **(Ai)** Left, diagram depicting the recording configuration; right, representative extracellular recording of a DP nerve in an isolated ganglion (G0) during a dopamine-induced *crawling* episode. **(Aii)** As in **(Ai)** for a chain of three ganglia (G1-G2-G3) where a DP nerve was recorded in each one. **(B)** Box plots describing the cycle frequency, duty cycle and firing frequency in G0, G1, G2, and G3; *n* = 12 ganglia from 11 leeches for G0, 15 ganglia from 11 leeches for G1, 16 ganglia from 14 leeches for G2, 13 ganglia from 10 leeches for G3. Comparison of G0 vs. G2 was performed by Wilcoxon rank-sum test; ^#^ indicates *p* = 0.027. For comparison of G1, G2, and G3 we used Kruskal-Wallis test; *p* > 0.05 for the three variables. **(C)** Dot plot describing the coefficient of variation (CV) of the cycle frequency, duty cycle and firing frequency in G0 and G2 [*n* as in panel **(B)**]. Each dot presents the CV value of the 10 cycles analyzed in each preparation. ^#^ indicates *p* < 0.001 (Wilcoxon rank-sum test).

Data analysis was performed using custom written Python codes. Spikes in DP nerves were detected using amplitude threshold. The motor pattern in both preparations was characterized by the cycle frequency (inverse of the time elapsed between the first DE-3 spikes in two successive bursts); the DE-3 duty cycle (calculated as the duration of each burst over the corresponding period); and the firing frequency of DE-3 (calculated as the number of spikes in a burst divided by the burst duration). In the experiments presented in Figure 2 we evaluated the first 10 cycles in single ganglia or the time interval that comprises the first 10 cycles in the anterior ganglia in the chain. Since dopamine initially elicited a long barrage of spikes, we considered the first cycle as the first of at least four consecutive cycles that fulfilled the following rule: cycle period between 4 and 45 s, burst duration between 2 and 22.5 s and duty cycle between 0.15 and 0.85.

To evaluate the effect of NS on the rhythmic inhibitory post-synaptic potentials recorded in the sensory T cells we identified the IPSPs and calculated the frequency of IPSPs before, during and after the pulse. The T cell traces were subjected to a Gaussian low-pass filter at 1 Hz that served to subtract the slow baseline variations. The trace resulting from this subtraction was filtered again with the same Gaussian low-pass. The IPSPs were recognized using an amplitude threshold that identified the negative peaks that were larger than 40% the maximal IPSP amplitude in each trace.

## Results

### Coordinated *Crawling* in a Reduced Preparation

We addressed the study on intersegmental coordination in the leech crawling motor pattern in a reduced preparation. Given that each segmental ganglion contain the network capable of generating the *crawling-pattern*, we asked whether three-ganglion chains could produce coordinated *crawling*, and if so, how it compares with that in single isolated ganglia.

The motor pattern in both preparations was monitored through the activity of the motoneuron DE-3 in extracellular recordings of the DP nerve (Figure 1B), corresponding to the contraction phase of crawling (Figures 1Aiii,iv). Figure 2A shows a representative example of dopamine-induced activity in a single ganglion (Figure 2Ai) and in each of the three ganglia of a chain (Figure 2Aii). The monoamine evoked rhythmic bursts of DE-3 in both experimental configurations. Figure 2B summarizes the cycle frequency of the *crawling-pattern*, and the DE-3 duty cycle and spike frequency in single ganglia (G0) and in middle ganglion of chains (G2). Figure 2C summarizes the coefficient of variation of these variables for each one of the preparations. These results show that the characteristics of the *crawling*-*pattern* were highly similar in both configurations, although the spike frequency in G2 was about 30% higher than in G0; and the coefficient of variation of the former was significantly larger than the latter. The larger firing frequency could indicate that the motoneurons are subjected to a larger basal input in the chain than in the isolated ganglion. Figure 2B also compares the activity of the three ganglia in the chain, showing that the motor pattern was markedly homogeneous among them.

**FIGURE 3:**
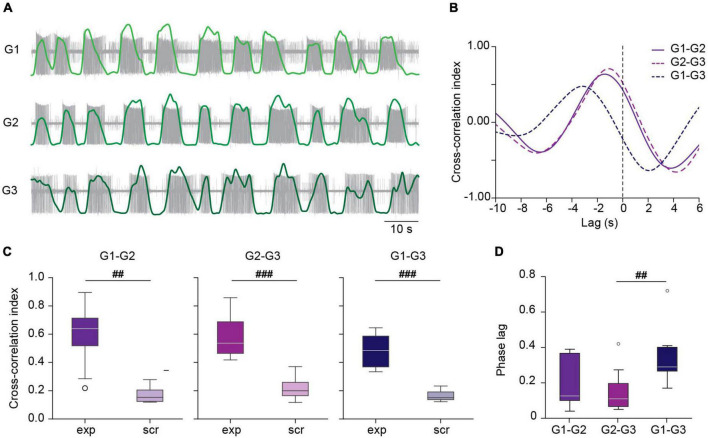
Correlated activity among ganglia during *crawling*. **(A)** Filtered versions of DP activity in a chain of ganglia (light, medium and dark green traces) superimposed on the original extracellular recordings (in gray) during a *crawling* episode. **(B)** Cross-correlation of the activity between the DP pairs shown in panel **(A)**. **(C)** Box plots comparing the cross-correlation index in experimental (exp) and scrambled (scr) nerve pairs. ^##^ indicates *p* < 0.005 and ^###^*p* < 0.001 (Wilcoxon rank-sum test); *n* = 8 chains from 7 leeches for exp and 8 recording combinations for scr. **(D)** Box plots showing the phase lag of each of the experimental nerve pairs. Friedman test *p* < 0.005, ^##^*p* < 0.01 (Conover’s *post-hoc* test). *n* = 8 chains from 7 leeches.

As observed in the example shown in Figure 2Aii the activity in the three ganglia of the chain was coordinated and exhibited a metachronal order. To assess the degree of correlation in the chains we performed a cross-correlation analysis of the activity in the three DP nerves. To this end, DP nerve recordings were rectified, filtered (Gaussian filter, sigma = 2,000), and decimated (10:1) (Figure 3A). Figure 3B shows the cross correlograms of the three recording pairs shown in panel A. As expected the G1–G3 lag doubles that between adjacent pairs (G1–G2 and G2–G3).

The peak cross-correlation index in all pairs was around 0.6 (Figure 3C). To test that these results were not due to mere chance we generated “scrambled” chains, combining a G1, G2, and G3 recording from different chains, preserving their location order. Comparison of the correlation index in experimental chains and in scrambled chains shows that in the latter cross-correlation is negligible (Figure 3C), suggesting that the correlation was due to a true coordinated activity among the crawling circuits in the chain. Figure 3D summarizes the phase lag (lag relative to the period) between the different ganglia in the chain as reported by the cross-correlation analysis. The results indicate a median phase lag for the DE-3 bursts of about 0.12 per segment.

These results clearly show that isolated chains of three ganglia exhibited *crawling* with metachronal order. This observation reveals the existence of a coordination mechanism independent of descending commands or peripheral feedback.

### Interaction Among Segmental Central Pattern Generators

Since the premotor NS neurons are linked locally to the CPG that controls the *crawling-pattern* (Figure 1C) we considered that manipulation of the NS membrane potential in the middle ganglion of the chain could serve as a means to evaluate the interactions among ganglia in the chain. While moderate hyperpolarizing current pulses (−0.5 to −2 nA) in NS decrease the cycle frequency of the *crawling-pattern* and the firing frequency of DE-3, strong hyperpolarizing pulses (−5 nA) shut down the activity completely in the isolated ganglion (Rodriguez et al., 2012). If the intersegmental coordination depended on elements extrinsic to the CPG that controls the *crawling-pattern* (Figure 1Di) we would expect the NS hyperpolarization to strongly reduce the rhythmic pattern locally, without affecting it in adjacent ganglia. Alternatively, if the coordination depended on the members of the CPG that controls the *crawling-pattern* (Figure 1Dii) we would expect the perturbation of the rhythmic pattern to spread through the chain.

**FIGURE 4:**
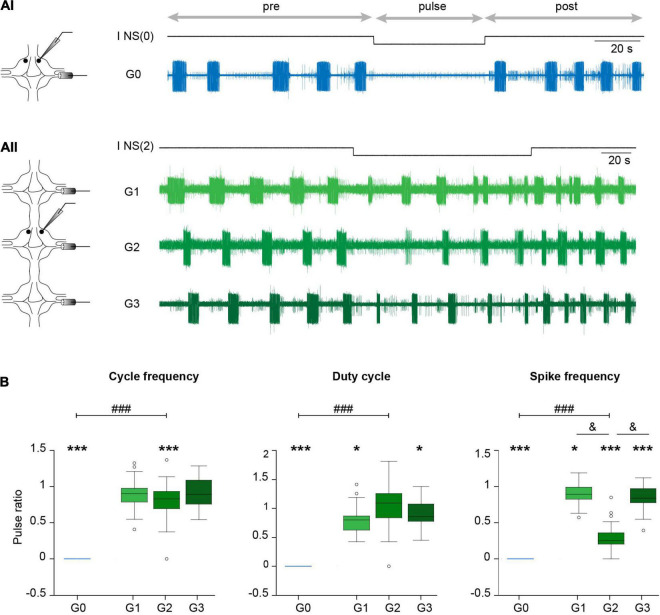
Effect of NS neuron upon *crawling*. **(Ai)** Extracellular recording of a DP nerve in an isolated ganglion during a *crawling* episode, while a square –5 nA current pulse was injected in an NS neuron [I NS(0)]. Horizontal arrows indicate the intervals considered in the analysis. **(Aii)** As in **(Ai)** for a chain of ganglia where a DP was recorded in each ganglion and the pulse was applied in one NS of G2 [I NS(2)]. **(B)** Box plots describing the relative cycle frequency, duty cycle and spike frequency in G0, G1, G2, and G3. *n* = 11 pulses in 7 *crawling* episodes in 6 leeches for G0; *n* = 18 pulses in 9 *crawling* episodes in 8 leeches for G1; 27 pulses in 12 *crawling* episodes in 10 leeches for G2; and 24 pulses in 10 *crawling* episodes in 7 leeches for G3. One sample Wilcoxon signed-rank test was applied to evaluate whether the values were different than 1. *** indicates *p* < 0.001 and * indicates *p* < 0.05. Comparison of G0 vs. G2 was performed by Wilcoxon rank-sum test; ^###^ indicates *p* < 0.00001. For comparison of G1, G2, and G3 we used Kruskal-Wallis test; *p* > 0.05 for cycle frequency and duty cycle and *p* < 0.00001 for cycle frequency; & indicates *p* < 0.00001 (Dunn’s *post-hoc* test).

**FIGURE 5:**
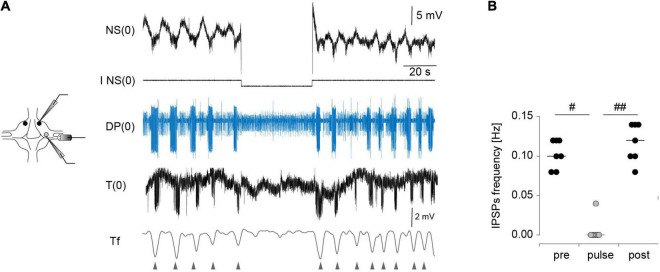
Effect of NS neuron upon the rhythmic activity of T cells during *crawling*. **(A)** Extracellular recordings of a DP nerve, and intracellular recordings of NS and T cells in an isolated ganglion, while a square –5-nA current pulse was injected in the NS neuron in the course of a *crawling* episode. The lowest trace is a filtered version of the T cell recording (Tf). The triangles indicate the timing of the IPSPs. **(B)** Frequency of the IPSPs measured before, during and after (pre, pulse, post, respectively) the pulse was applied. *p* < 0.01 for comparison of pre, pulse and test (Friedman test), ^#^ indicates *p* < 0.05, and ^##^*p* < 0.01 (Conover *post-hoc* test). *n* = 7 pulses in 5 *crawling* episodes in 4 leeches.

Application of a −5-nA pulse to one NS in isolated ganglia reversibly abolished the activity of DE-3 during the *crawling-pattern* (Figures 4Ai,B). To prove that the effect of the hyperpolarizing pulses in NS were not only due to the direct inhibition of the motoneurons through the rectifying junctions (Figure 1C) but to the inhibition of the CPG, we used an alternative proxy of the *crawling-pattern*. Mechanosensory T cells are subjected to a reafferent inhibitory signal from the CPG (Figure 1C). If NS was effectively linked to the CPG that controls the *crawling-pattern*, we expected that not only DE-3 bursts were abolished during the NS hyperpolarization but the T IPSPs too. Figure 5A shows that T cells exhibit rhythmic IPSPs in phase with DE-3 bursts in the course of the *crawling-pattern* and that the injection of a −5-nA pulse suppressed both rhythmic signals (Figures 5A,B). This result indicates that strong hyperpolarizing pulses in NS reversibly inhibited the CPG that controls the *crawling-pattern*.

Surprisingly, the same pulses that completely eliminated the rhythmic activity in the isolated ganglia (Figures 4A, 5) had almost no effect in the three-ganglion chain. Application of −5-nA pulses to NS cells in the middle ganglion slightly slowed down the rhythm (Figure 4Aii) but the pattern was clearly present. Doubling the current intensity to −10 nA did not produce further reduction in the rhythm (*p* > 0.05 Wilcoxon rank-sum test; *n* = 5–9 pulses in 5–7 episodes in 4–6 leeches for −5 nA and *n* = 11–19 pulses in 7–9 episodes in 6–8 leeches for −10 nA).

To quantify the effect of NS hyperpolarization the variables are expressed as the ratio between the value measured during the pulse and the control, where the latter was calculated as the mean measured in four cycles before and after (pre and post, respectively) the pulse. Two comparisons were made: between G0 and G2; and among the G1, G2, and G3. The first evaluates the influence of interganglionic interactions, in contrast with the isolated ganglion. The second evaluates the spread of the NS influence along the chain.

The effects of NS hyperpolarization in the middle ganglion of the chain were drastically smaller than in the isolated ganglion (Figure 4B). The cycle frequency of the *crawling-pattern* in G2 was only reduced to about 80% the control value; the DE-3 duty cycle was unaltered. In contrast, NS hyperpolarization reduced the firing frequency of DE-3 in G2 to 30% the control value. Thus, the motoneuron activity was the most affected variable in G2, but still markedly less than in G0.

Analysis of the impact that the NS hyperpolarization exerted on adjacent ganglia in the chain (Figure 4B) shows that the small local (G2) effect on cycle frequency was not reflected in adjacent ganglia (G1 and G3), while the marked local effect on the firing frequency of DE-3 was transmitted to the anterior and posterior ganglia, but to a lesser degree. Surprisingly, the duty cycle was slightly reduced in adjacent ganglia, while no effect was observed locally.

Taken together these results might cast aside the hypothesis that the coordinated activity in the chain results from the coordination of autonomous segmental networks.

### Extent of Influence of the Premotor Non-Spiking Neuron in the Chain

To support the interpretation stated above it is important to test that the properties of the premotor NS neurons in the chain are similar to those observed in the single ganglion, and thus their ability to affect their synaptic targets.

To evaluate this question, we compared the effect that hyperpolarizing NS pulses exerted on basal motoneuron activity in DP nerves of isolated ganglia and of three-ganglion chains. Motoneurons are connected to NS through rectifying electrical junctions (Figure 1C), and therefore it is expected that if the premotor neuron properties in the chain differ from those in isolated ganglia, the effectiveness of the interaction between NS and the motoneurons should differ too.

**FIGURE 6:**
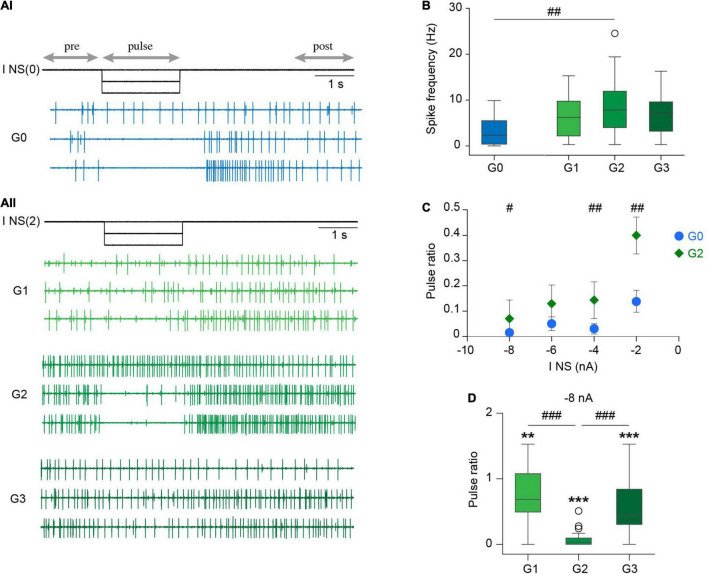
Effect of NS upon DP basal activity. **(Ai)** Extracellular recordings of spontaneous DP activity performed in an isolated ganglion while square hyperpolarizing current pulses of increasing amplitude were injected in the NS neuron [I NS(0)]. For simplicity we only show 0, –4, and –8 nA current pulses (upper, middle and lower trace, respectively). Horizontal arrows indicate the intervals considered in the analysis. **(Aii)** As in **(Ai)** for a chain of ganglia; the current was injected in one NS neuron of the middle ganglia [I NS(2)]. **(B)** Box plots comparing DP basal spike frequency in isolated and chain ganglia. ^##^ indicates *p* < 0.002 for G0 vs. G2 (Wilcoxon rank-sum test). *p* > 0.05 (Kruskal-Wallis test) for G1, G2, and G3. *n* = 20 ganglia from 12 leeches for G0, 26 ganglia from 16 to 17 and 16 leeches for G1, G2, and G3, respectively. **(C)** Scatter plot showing the pulse ratio in isolated and middle ganglia for hyperpolarizing pulses of increasing amplitude. All points are significantly different than 1 (*p* < 0.001, One sample Wilcoxon signed-rank test against 1); ^#^ indicates *p* = 0.02 and ^##^ indicates *p* = 0.002 (Wilcoxon rank-sum test). *n* = 17–19 ganglia from 11 leeches for G0 and 24–25 ganglia from 15 to 16 leeches for G2. **(D)** Box plots of pulse ratio in chains of ganglia for –8 nA hyperpolarizing pulses applied in G2. ** indicates *p* < 0.01, ***p < 0.0001 (One sample Wilcoxon signed-rank test against 1). *p* < 0.00001 for comparison of G1, G2, and G3 (Kruskal-Wallis test), ^###^*p* < 0.001 (Dunn’s *post-hoc* test). *n* = 22 ganglia from 13 leeches for G1, 25 ganglia from 16 leeches for G2 and 24 ganglia from 14 leeches for G3.

First, it is to notice that the basal activity of DP nerves of the isolated ganglion (Figure 6Ai) was lower than that in the middle ganglion of the chain (Figure 6Aii). In average, the basal firing frequency in the DP nerves of G2 was about 160% higher than that of G0, while the three ganglia in the chain showed similar basal activity (Figure 6B). This result suggests that the motoneurons were subjected to a higher excitatory drive in the chain than in the isolated ganglion.

**FIGURE 7:**
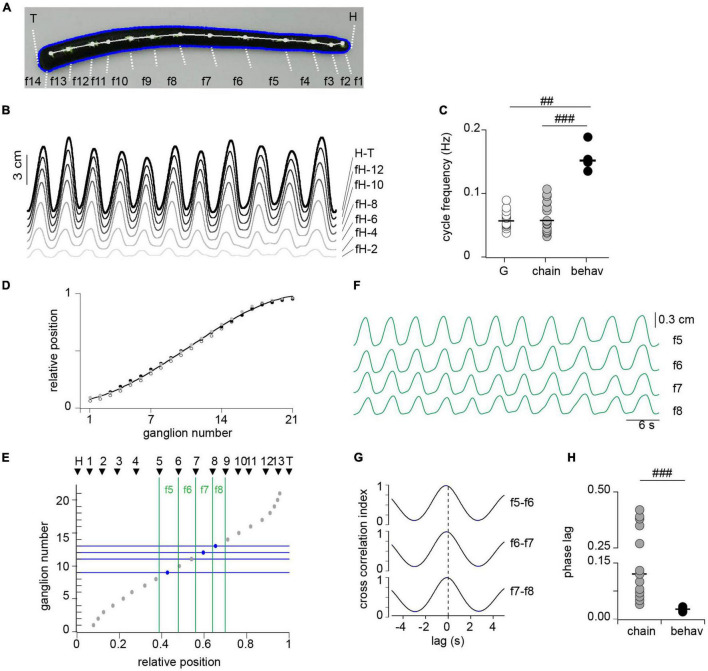
Correlated activity among segments during crawling. **(A)** Snapshot from a video during the elongation phase of crawling. Thirteen white dots were painted over the dorsal midline determining 14 fragments (f1–f14) along the longitudinal axis, including the head (H) and tail (T) edges as markers. **(B)** Length change of fragments between head edge (H) to successive dots, including the head to tail (H-T). For clarity we show every other fragment. **(C)** Cycle frequency measured in isolated ganglia (G), in three-ganglion chains and in intact animals (behav); *n* = 12 ganglia from 11 leeches (G), 16 chains from 14 leeches (chain) and 4 intact animals (behav). ^##^ indicates *p* < 0.005 and ^###^ indicates *p* < 0.0005 (Wilcoxon rank-sum test). **(D)** Relative position of midbody ganglia 1–21; each symbol represents one leech (*n* = 3). The line presents a polynomial fit (*R* = 0.99). **(E)** The gray circles show the ganglion number as a function of the mean relative position along the leech longitudinal axis; and the black triangles show the relative position of each of the points drawn on the leech shown in panel **(A)**. **(F)** Length changes of fragments f5–f8. **(G)** Cross-correlation of pairs of traces shown in panel **(F)**, identified on the right. **(H)** Phase lag measured in isolated three-ganglion chains and in the animal. *n* = 8 chains from 7 leeches for chains, and 4 intact animals for behav. ^###^ indicates *p* < 0.001 (Wilcoxon rank-sum test).

Hyperpolarizing pulses of increasing amplitude (0 to −8 nA) strongly reduced the basal spontaneous DP activity in the isolated ganglion (Figure 6Ai) and in the middle ganglion of the chain (Figure 6Aii). To quantify these effects the results are expressed as the ratio between the firing frequency during the pulse and the control [calculated as the mean of the frequency before (pre) and after (post) the pulse].

Inhibitory pulses of low amplitude (−2 nA) reduced the basal motoneuron activity in G0 and in G2, but the effect was markedly larger in the former (Figure 6C). Pulses of larger amplitude, instead, produced a more similar effect in both configurations (Figures 6A,C). Considering that the basal activity in the chain was higher than in the single ganglion, the results suggest that the properties of the premotor NS neuron and its synaptic connectivity in the chain and in isolated ganglia were similar. Therefore, the differential effect of NS on the *crawling-pattern* in both experimental configurations cannot be based on this factor.

Surprisingly, hyperpolarization of NS in the middle ganglion exerted inhibitory effects on motoneurons of adjacent ganglia, albeit at a much lower degree (Figure 6D). The neuritic arborization of motoneurons is confined within the corresponding segment (Stuart, 1970; Fan et al., 2005), while NS extends neurites through the anterior and posterior connectives (Wadepuhl, 1989). If NS were to exert its effect directly on motoneurons one ganglion away, the hyperpolarizing signal should have spread passively over a distance of around 500 μm. As this is an unlikely possibility we propose an alternative setting: motoneurons are under the excitatory influence of a cord spanning interneuron that is tonically active, and is inhibited by NS hyperpolarization. One should take in consideration that neuronal projections in the leech remain viable even in the absence of their soma for several hours (Van essen and Jansen, 1977). Inhibition of the excitatory interneuron would mediate the inhibitory effect of NS on motoneurons in adjacent ganglia, while locally the NS would act, in addition, through the electrical synapse.

### Comparison of the Motor Pattern in Reduced Preparations and in Intact Animals

To contrast the motor pattern generated *ex vivo* (in single or chain of ganglia) with that produced *in vivo* we implemented kinematic measurements in intact animals during crawling. Crawling was monitored as changes in length of the whole animal, and of fragments defined by dots painted along the dorsal longitudinal axis of the leech (Figure 7A). The cycle frequency was measured as the inverse of the time elapsed between two successive peaks of the whole animal length, considering the average of the first 11 recorded oscillations (Figure 7B, H-T trace). The cycle frequency measured in this way was about three times that measured in the isolated ganglia or chains (Figure 7C).

To estimate the intersegmental lag in the whole animal it was necessary to relate the fragments with anatomical segments. Because ganglia are landmarks of segmentation we evaluated whether their relative position along the animal can be used as a marker. To this end we dissected animals, exposing the complete chain of ganglia within their stretched body and estimated the relative location of each ganglion along the antero-posterior axis of the animal (Figure 7D). The results show that ganglia are located in highly conserved positions relative to the longitudinal axis and therefore the relative distance from the head can be used to identify segmental location.

To match the position of each dot relative to the total length of the animal we proceeded as follows: (a) the length from the head up to each dot was measured during a crawling episode (Figure 7B); (b) each length trace was divided by the total length (trace H-T in Figure 7B) to obtain a “relative length trace”; and (c) the latter was averaged along the crawling episode to obtain the relative position of each dot. Figure 7E presents the relative positions of the dots thus measured (black triangles), corresponding to the animal shown in panel A, on a graph that depicts the ganglion number as a function of the relative position along the longitudinal axis (inversion of Figure 7D). To compare the data obtained *in vivo* with that obtained *ex vivo* we focused on the fragments that correspond to the range of segments (7–13) included in the electrophysiological studies (green vertical lines in Figure 7E). Figure 7F displays the length changes of the four fragments identified in the leech shown in panel A, and Figure 7G displays the cross-correlation of these traces. These middle fragments show a similar high correlation among each other. The average cross-correlation index thus measured in the four studied animals was 0.97 ± 0.006 (mean ± SEM). To calculate the phase lag per segment we considered that the timing of the peak elongation (or contraction) of each fragment was determined by the most posterior ganglion contained in it (horizontal blue lines in Figure 7E). Assuming these considerations the results show that the intersegmental phase lag was of about 0.03 per segment, a value that is markedly shorter than that measured for DE-3 in the three-ganglion chains (Figure 7H).

These results show that while *in vivo* the cycle frequency develops in the same order of magnitude than *ex vivo*, the intersegmental phase lag in the whole animal is a fourth of that measured in the chain of ganglia. These observations suggest that the CPG in the whole animal operates within a similar temporal constrain than *ex vivo*; but the signals that grant coordination of the DE-3 activity are transmitted slower than those that grant segmental coordination *in vivo* (e.g., multiple parallel pathways operate in the latter, including proprioceptive feedback).

## Discussion

The analysis of fictive motor behaviors has been studied in isolated nervous systems of different animals (Hill et al., 2003). Leeches offer a favored context to this analysis because of the uniformity of the body segments and the ganglia that innervate them, freeing the analysis from the possible role of segmental specializations. As a neuronal correlate of crawling can be evoked in isolated midbody ganglia we addressed the study of intersegmental interactions in short chains of ganglia, devoid of descending signals and peripheral feedback. An isolated chain of three midbody ganglia exhibited coordinated *crawling*, that comply with the antero-posterior order observed *in vivo*, which is a clear indication of an inter-ganglionic mechanism of coordination. The hyperpolarization of a premotor neuron that suspended the motor pattern in isolated single ganglia, only produced a slight effect on the motor pattern in chains of three ganglia. Taken together these observations indicate that: peripheral feedback is not necessary for the observed correlation; and although each segmental ganglion contains all the elements necessary to produce the *crawling*-*pattern*, the network resulting from the interactions within a chain of ganglia cannot be considered as a series of independent modules coordinated by an extrinsic element. If, as suggested by the hypothesis presented in Figures 1Di, coordination depended on an element extrinsic to the CPG, local perturbation would have prevailed. The fact that the local perturbation markedly lost its power indicates that coordination resulted from a global network in which interactions among adjacent ganglia, as schematically represented in Figures 1Dii, turns the system refractory to local perturbations. In fact the results contemplate the involvement of long-range intersegmental interactions as observed in lamprey (Ayali et al., 2007) and in leech swimming (Pearce and Friesen, 1985). Moreover, it is possible that cord-spanning cephalic neurons, that have been identified as a context-dependent command neuron for swimming and *crawling* (Esch et al., 2002), and whose activity modulates the latter (Puhl et al., 2012), can be part of the intersegmental coordination described here.

### Premotor Non-spiking Neuron in Crawling

Non-spiking neurons play in the leech a role similar to that of the spinal Renshaw (1941) cells of vertebrates. These premotor neurons are at the center of a recurrent inhibitory circuit that modulates motor output (Renshaw, 1941; Alvarez and Fyffe, 2007; Szczupak, 2014), and here we revealed that the inhibitory effect of the premotor NS neuron is not confined to local motoneurons. We interpret that motoneurons are under the influence of cord-spanning pathway (composed by a single or several neurons) that exerts a positive drive, through which NS can influence motoneurons in, at least, adjacent ganglia. This effect was observed at basal conditions and during *crawling*.

Previous work proposed that the premotor NS neuron is not only directly linked to the motoneurons but is also linked to the rhythmogenic circuit (Figure 1C). Here we further support the connection with the CPG by showing that inhibitory pulses in NS not only canceled the motoneuron bursting, but it also canceled the corollary discharge that the CPG impinges onto the mechanosensory T cells (Alonso et al., 2020).

While NS preserved its influence on motoneuron firing in the context of the chain, its influence on the CPG was drastically diminished. We interpret that the local effect of NS on a(n) element(s) of the local CPG was weakened under the influence of intersegmental CPG interactions, while the direct action of the premotor neuron on the motoneurons was preserved.

### Quantitative Comparison With *in vivo* Measurements

The *crawling* rhythmic activity observed in the isolated chain of ganglia is compatible with the characteristics of crawling observed *in vivo* but exhibited quantitative differences. The *in vivo* experiments developed in the present work show a crawling period of around 5 s, that coincides with previous observations (Stern-Tomlinson et al., 1986; Cacciatore et al., 2000; Eisenhart et al., 2000). In isolated ganglia and in the chain of three ganglia the rhythmic pattern exhibited a period of around 10 s; this value is similar to that reported for experiments in which the whole cord was isolated (Eisenhart et al., 2000; Puhl and Mesce, 2010). Thus, in three different *ex vivo* configurations, with increasing levels of reductionism, the period was well preserved, and was larger than *in vivo*. These results suggest that peripheral signals may accelerate the rhythmic behavior, as already described in other animal models (Pulver et al., 2015; Fushiki et al., 2016).

The intersegmental phase lag measured in our *in vivo* experiments was of around 0.03 per segment; this value also coincides with previous publications (Stern-Tomlinson et al., 1986; Cacciatore et al., 2000; Eisenhart et al., 2000). In the three-ganglion chain, we measured a phase lag of around 0.12 per ganglia, which was larger than that obtained in experiments in which the whole cord was isolated (Eisenhart et al., 2000; Puhl and Mesce, 2010). Thereafter, the results suggest that the intersegmental delay in crawling is affected by peripheral feedback, descending signals, and probably further interganglionic signals. In support of the influence of intersegmental interactions on this variable it was observed that the intersegmental delay measured for leech swimming increases with the length of the chain of ganglia (Pearce and Friesen, 1985).

Taken together, published results and those presented here show that the network controlling crawling in the leech operates under several layers of control: each ganglion is endowed with the whole neuronal network that generates the rhythmic motor pattern (Puhl and Mesce, 2008); within the chain, interactions among ganglia form an emergent network that subdues possible local perturbations; a pair of command neurons in the brain initiate crawling and provide additional coordinating signals (Puhl et al., 2012); and sensory feedback probably modulates both the rhythm and the transmission of coordinating signals along the cord.

### Leech Crawling in the Context of Animal Rhythmic Behaviors

Intersegmental coordination in isolated nervous systems has been shown in different vertebrates and invertebrates, demonstrating that it can take place in the absence of peripheral feedback. Swimming in leech (Kristan et al., 2005), crayfish (Mulloney and Smarandache-Wellmann, 2012), and lamprey (Grillner and Wallen, 2002), and mammalian locomotion (Grillner and Wallen, 2002) comply with this principle. These studies suggest that while sensory feedback is a key modulator of actual behavior, intersegmental coordination takes place in the absence of peripheral inputs. Among insects, cockroach and stick insects provide contrasting examples: while in the former the isolated nervous system can generate a pattern compatible with walking, in the latter this only happens in the presence of peripheral feedback (Ayali et al., 2015). Our study on leech crawling is in line with the majority of these studies, indicating that motor behaviors result from multiple layers of control.

The present work puts forward strong evidence that intersegmental interactions can be considered a backbone of coordinated activity. Moreover, we show that the interactions among segmental modules engender a global network, and this reduces the degrees of freedom of the system because it subdues the autonomy of the modules. Work in zebrafish has suggested the idea of the spinal circuit functioning as a continuum (Wiggin et al., 2015), but what distinguishes the analysis presented here is that in the leech the segmental rhythmogenic network could operate autonomously and this autonomy was subdued in the context of the chain.

## Data Availability Statement

The raw data supporting the conclusions of this article will be made available by the authors, without undue reservation.

## Author Contributions

GK, ASM, and LS conceived and performed experiments, and analyzed the data. MR performed experiments and analyzed the data. GK and LS wrote the manuscript. All authors contributed to the article and approved the submitted version.

## Conflict of Interest

The authors declare that the research was conducted in the absence of any commercial or financial relationships that could be construed as a potential conflict of interest.

## Publisher’s Note

All claims expressed in this article are solely those of the authors and do not necessarily represent those of their affiliated organizations, or those of the publisher, the editors and the reviewers. Any product that may be evaluated in this article, or claim that may be made by its manufacturer, is not guaranteed or endorsed by the publisher.

## Footnotes

1
https://www.python.org/
